# Comparison of Students’ Self-Assessment and Simulated Patient Assessment in a Patient Counseling Evaluation and Perceived Importance of Communication Skills

**DOI:** 10.3390/pharmacy10060177

**Published:** 2022-12-19

**Authors:** Sara A. Wettergreen, Maria J. Pearson, Sarah K. Scoular

**Affiliations:** 1 Department of Clinical Pharmacy, Skaggs School of Pharmacy and Pharmaceutical Sciences, University of Colorado, Aurora, CO 80045, USA; 2 UCHealth Memorial Hospital, Colorado Springs, CO 80909, USA; 3 Skaggs School of Pharmacy, University of Montana, Missoula, MT 59812, USA

**Keywords:** pharmacy communication, empathy, self-assessment, simulated patient

## Abstract

The primary objective of this study was to compare students’ self-assessment ratings with simulated patient (SP) assessment ratings of communication skills in a patient counseling Objective Structured Clinical Exam (OSCE). The secondary objective was to evaluate student perceptions of the importance of communication skills in the practice of pharmacy as well as the impact of a virtual OSCE format. First-year pharmacy students completed an OSCE focused on self-care product counseling. The evaluation was graded using a rubric covering both verbal and non-verbal communication. Students who completed the course were provided a 15-question, post-evaluation survey with questions related to self-assessment of communication skills and perceptions of the importance of communication skills. Of the 138 students in the course, 68 completed the optional post-assessment survey (49% response rate). There were no statistically significant differences between the ratings by students and SPs for the four communication elements included in the self-assessment. Most of the students recognized the importance of communication skills, including developing rapport and trust. Recognition of the importance of communication skills to future practice as a pharmacist positively correlated with performance on the evaluation (r^2^ = 0.5409, *p*-value = 0.0007). Student self-assessment is an effective and cost-effective mode of feedback for practice experiences as an alternative to the use of SPs.

## 1. Introduction

The development of “soft skills”, such as interpersonal skills, communication skills, empathy, professionalism, and emotional intelligence, can be found within lesson plans and curricula of pharmacy schools across the nation [1,2]. Communication skills are a core element of effective patient care. As a result, the Accreditation Council for Pharmacy Education (ACPE) standard 3.6 notes that effective verbal and non-verbal communication skills are important for pharmacy graduates [3]. The importance of communication skills is further recognized through the Curriculum Outcomes and Entrustable Professional Activities (COEPA) published by the American Association of Colleges of Pharmacy [4]. The communication-related COEPA skills emphasize the specific soft skills of actively engaging, listening, and communicating verbally and non-verbally with individuals. Communicating clearly and effectively, demonstrating empathy, and showing compassion are more closely tied to strong patient care skills than ever before [5].

Communication skills can be taught and assessed through a variety of methodologies [6,7]. In healthcare education, performance-based assessments using either standardized or simulated patients have been used to assess communication skills [8,9,10,11]. While the terms standardized patient and simulated patient are often used interchangeably, a simulated patient (SP) describes an actor portraying a patient in a realistic way [12]. The use of SPs in pharmacy education is viewed as important to building communication and counseling skills, and helpful in preparing students to apply patient care skills in real life [13]. SPs are trained to provide a consistent portrayal of a scenario, complete assessment instruments, and share a unique feedback perspective [12]. These skillsets support the integration of SPs within performance-based assessments such as those for communication.

As SPs are trained in assessment, the results of their scoring of an assessment instrument can serve as a comparison for students’ self-assessment. Several studies have compared students’ self-assessment to ratings by a faculty observer or SP within communication-focused assessments; however, there are conflicting conclusions. Some studies showed a significant difference between students’ self-assessment and the ratings by an observer, and some studies did not show a difference. In a study by Pawluk and colleagues, student self-assessments were compared to both faculty and SP assessments of performance [14]. The 24 first-year pharmacy student participants had four different encounters with a SP, and self-assessments were performed for each station. This study found that first-year pharmacy students assessed their communication skills more positively than both faculty and SPs. Similarly, data from three meta-analyses conducted from 35 published articles on medical student self-assessment showed that students were more likely to overestimate performance on communication-based, SP encounters than knowledge-based evaluations [15]. In contrast, students scored their communication skills lower than the ratings of observers in a study that evaluated the accuracy of medical student self-assessment of communication skills [5]. Recently within pharmacy education, students’ self-assessment of communication skills was found to be similar to comparators. Lempicki and colleagues assessed pharmacy students’ communication self-evaluation skills by comparing student self-evaluations with those completed by course graders and SPs [16]. The study found a high level of agreement observed between communication skills evaluations completed by students, course graders, and SPs. The study also postulated that self-evaluation of communication skills may be an acceptable alternative to faculty or SP evaluations when appropriate.

There is limited data comparing student and observer ratings for specific communication skills such as the ability to demonstrate empathy. In a study by Murry and colleagues, there was a positive association between the scores on communication rubrics and student empathy categorization [17]. Another finding of this study was that SPs frequently provided empathy feedback to students, which suggested that empathy was important to the patient encounter. 

Upon receipt of a Doctor of Pharmacy degree, there remains a need for continuous professional development of both clinical and non-clinical skills. In fact, it could be argued that, outside of a setting that requires regular feedback and assessment, the ability to self-assess becomes even more important. With the knowledge that one’s aptitude for self-assessment can influence the trajectory of their career, having a working knowledge of the accuracy of students’ self-assessment is crucial. Objective Structured Clinical Examinations (OSCEs) play an important role in this arena. It is critical that pharmacy students and pharmacists can effectively communicate their knowledge, as this is an essential component of ensuring advanced pharmacy practice experience-, practice-, and team-readiness [18].

The studies mentioned above make it difficult to ignore the importance of communication, empathy, and metacognitive abilities in the development of future pharmacists. There is a paucity of information related to the assessment of soft skills within patient-centered communication. More studies are needed to make a strong conclusion about how students’ self-assessment compares to that of an observer within communication soft skills. Evaluating student perceptions of the importance of communication skills as well as assessing the reliability of student self-grading of these skills versus SPs in the virtual environment gives more information regarding student buy in and explores a financially feasible alternative for pharmacy schools to conduct practice-based assessments using student self-evaluation, both in person and virtually, in order to improve access to OSCE based evaluations when there are limited resources.

This study aims to determine if a student self-assessment is comparable to that of a SP regarding communication abilities. Additionally, it aims to elucidate student perceptions of the importance of communication in the practice of pharmacy.

## 2. Materials and Methods

As part of the University of Colorado Skaggs School of Pharmacy and Pharmaceutical Sciences program, first-year students take a required Patient-Centered Communication course. In this course, students complete three OSCEs focused on communication skills. This study was related to an OSCE focused on providing patient counseling on a self-care product to a SP in a single, one-on-one, 10-min encounter that took place via Zoom. This is the second communication OSCE of the course and includes a practice evaluation to familiarize students with the process. During the virtual OSCE, the SP grades the student’s performance on both the completeness of the information communicated during counseling as well as the verbal and non-verbal communication skills demonstrated using a rubric. The rubric includes 19 patient-counseling components consisting of greeting the patient appropriately, providing a roadmap of the interaction, verification of patient understanding through “teach-back” methods, demonstrating empathy and using appropriate non-verbal behavior, as well as using patient-friendly language and attending to timing. Since this OSCE focused on counseling on a self-care product, additional components, such as describing the expected benefit of the product, special administration instructions, relevant side effects and how to prevent or manage these side effects, advising the patient of symptoms that require further medical attention, and how to properly store the product, were also included in the rubric. The rubric was graded on a point scale with points distributed for full credit, partial, or no credit. There were 138 students enrolled in the course during the survey timeframe in the fall of 2020.

The self-assessment data were collected by providing students with an optional survey after the evaluation, which is available in the Supplementary Materials. The post-evaluation survey was created by research team members and included a self-assessment of performance on four communication components. These four components with a brief summary are: empathy (acknowledgment of the patient’s experience verbally and non-verbally); checking in with the patient to assess their understanding, which we refer to as “practicing chunks and checks for understanding”; appropriate non-verbal communication (eye contact, facial expression, vocal rate and tone, use of fillers conveying confidence), and use of patient-friendly language (avoiding medical jargon). Practicing chunks and checks for understanding was rated as either yes or no, while empathy, appropriate non-verbal communication, and use of patient-friendly language were rated as either yes, partially, or no. The students were trained on the performance criteria in the rubric through in-class demonstrations and practice experiences including a practice evaluation with feedback from the SPs. The SPs were trained on rubric performance criteria in a one-hour training session prior to the evaluation. The SPs were also instructed to provide comments if a rating other than “yes” was selected. The second portion of the survey included questions to assess student perceptions of their performance as well as the importance of communication skills to the practice of pharmacy.

The patient counseling encounter was recorded by the student during the evaluation to allow for self-assessment. The survey was distributed through Qualtrics. A cover page (enclosed in the same document as the survey) was displayed on the first webpage of the electronic survey, which outlined any (or lack thereof) conflicts of interest and served to obtain participant consent. The survey was self-administered, consisted of 15 questions, and took approximately 15 min to complete. The survey consisted of demographics, a self-assessment of performance in the patient counseling encounter, and perceptions of their performance in the encounter and of the importance of communication skills to the practice of pharmacy. The students were instructed to review the video recording of their evaluation prior to completion of the survey. The survey window was open for one week immediately following the evaluation, before grades or SP feedback were available.

The primary objective of this study was to compare the students’ self-assessment ratings with the SP assessment ratings of communication skills in a patient counseling OSCE. The secondary objective was to evaluate student perceptions of the importance of communication skills in the practice of pharmacy as well as the impact of a virtual OSCE format.

This research was submitted to the Colorado Multiple Institutional Review Board (IRB) and deemed exempt from IRB review. Statistical analysis was performed using SPSS. Tests of proportions between categorical outcomes were performed using Fisher’s exact test. Cramer’s V was used for determining correlation. Tests of correlation between continuous variables and ordered categorical variables were analyzed using polyserial correlations with chi-square tests for significance.

## 3. Results

### 3.1. Demographics

Of the 138 students in the Patient-Centered Communications course in the fall of 2020, 68 students completed the optional post-assessment survey (49% response rate). The mean participant age was 26 years, and approximately 70% of the participants were female. Additional demographics are listed in Table 1.

### 3.2. Comparison of Students’ Self-Assessment and Simulated Patient Assessment

There were no statistically significant differences between the ratings by the students and SPs for the four communication elements that were included: completing chunks and checks, demonstrating empathy, using patient-friendly language, and non-verbal communication skills, as seen in Table 2. Most of the students received a full-credit score for the items of completing chunks and checks and non-verbal communication skills. Demonstrating empathy was the lowest-rated item by the SPs (60.3% yes), which was equally rated by the students’ self-assessment (60.3% yes). Non-verbal communication was the lowest-rated item by the students’ self-assessment (55.9% yes).

### 3.3. Evaluation Performance Satisfaction

**Figure 1:**
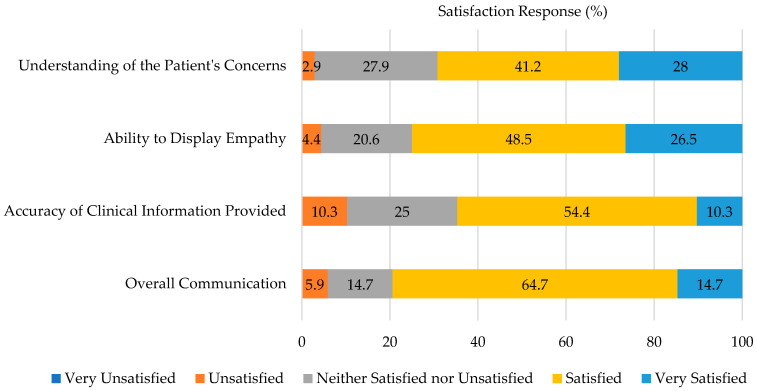
Evaluation Performance Satisfaction.

When self-assessing their own performance, the students were satisfied overall with their communication skills in the patient counseling OSCE (79.4% satisfied or very satisfied), as seen in Figure 1. More students were satisfied with their ability to display empathy compared to their ability to provide accurate clinical information (75% versus 64.7%, respectively).

### 3.4. Perceptions of Communication Skills

**Figure 2:**
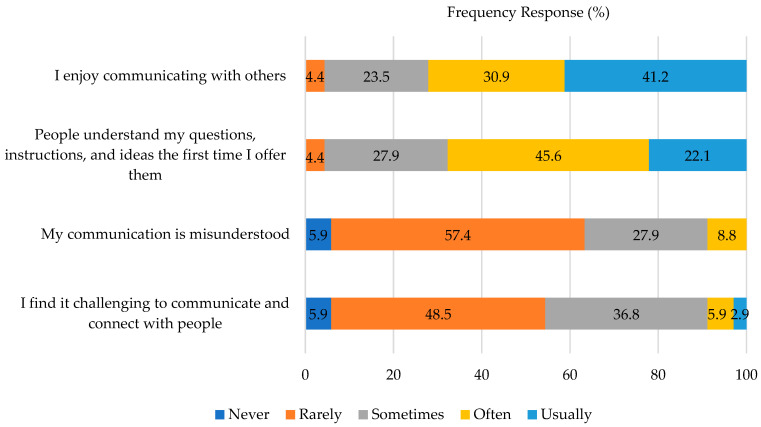
Communication Self-Perceptions.

The students generally demonstrated positivity toward their experiences communicating with others (Figure 2). All of the students noted that they enjoy communicating with others at least some of the time, while some experienced this more often than others. A majority of the students noted that they are rarely misunderstood, and that their ideas are understood the first time they are offered.

The development of soft skills was viewed as important by 92.7% of the students (Table 3). Nearly all of the students recognized the importance of communication skills for future practice (97.1%) and felt communication was important to developing patient rapport and trust (95.6%). There was no correlation between the SP ratings of empathy and the students’ recognition of the importance of the development of soft skills such as empathy (r^2^ = 0.3159, *p*-value = 0.107). Student recognition of the importance of soft skills positively correlated with OSCE performance (r^2^ = 0.4667, *p*-value = 0.0011). Recognition of the importance of communication skills to future practice as a pharmacist positively correlated with OSCE performance (r^2^ = 0.5409, *p*-value = 0.0007) and the final grade in the communications course (r^2^ = 0.4404, *p*-value = 0.0112). Additionally, recognition of the importance of using communication to develop patient rapport and trust positively correlated with OSCE performance (r^2^ = 0.5469, *p*-value = 0.0012) and the final grade in the communications course (r^2^ = 0.5405, *p*-value = 0.0007).

Additionally, a majority of the students noted that it was more important to communicate well than to be knowledgeable (79.4% vs. 20.6%, respectively). The students also felt it was more important to listen than to be heard (94% vs. 6%, respectively).

### 3.5. Perceptions of Virtual Evaluation Format

**Figure 3:**
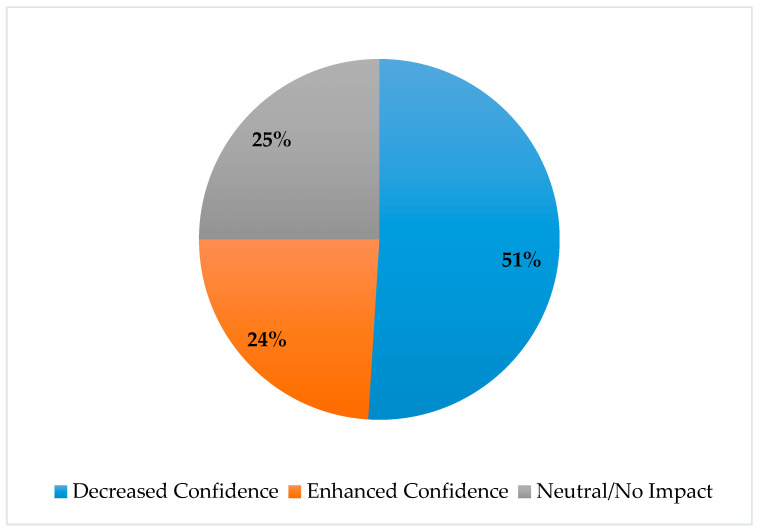
How did the virtual format of the evaluation affect your confidence in the encounter?

A majority of the students (51%) felt that the virtual format of the evaluation hindered their confidence in the encounter, while 24% of the students felt the virtual format enhanced their confidence (Figure 3).

Half of the students felt the virtual format of the evaluation had no impact on their ability to provide empathy (50%) or develop a trusting relationship (50%) with the patient (Table 4). However, 41.2% and 45.6% of the students felt the virtual format hindered these two abilities, respectively. The majority (60.3%) of the students felt the virtual format hindered their ability to demonstrate appropriate non-verbal communication.

## 4. Discussion

Overall, the students’ self-assessment was comparable to the SP ratings for four specific communication skills: completing chunks and checks, demonstrating empathy, using patient-friendly language, and non-verbal communication skills. Similar findings were recently observed in a study by Lempicki and colleagues, where there was agreement between student, SP, and faculty ratings in a communication assessment in pharmacy education [16]. However, other studies have noted differences between SP and student self-assessment ratings of communication skills [14,19,20]. Students are often concerned that SP evaluations are overly critical, based on our experiences as communications course directors. This is challenging because of the inherently subjective nature of the evaluation of soft skills such as demonstrating empathy. Comparable ratings between SPs and student self-assessments of communication skills supports the usefulness of SP ratings as an indicator of performance. The similarity in ratings also supports that students may be able to self-assess performance in practice scenarios where SPs may not be available or when funding limits additional practice evaluations with SPs. At the University of Colorado Anschutz Medical Campus, SPs undergo extensive training within their roles, including evaluation-specific instruction and modeling, by course faculty. It is unknown if similar results would be seen in an environment where there is less SP training or where faculty or staff are used as simulated patients. It is also important to note the difference between simulated patients who are actors portraying a patient with a history facilitated by the director of the assessment and standardized patients who are real patients with their own medical, social, and psychological history [21]. It is unknown whether similar observations would be seen in OSCEs where standardized patients are used.

When comparing the four communication skills assessed, empathy was the lowest rated item by the SPs, with only about 60% of the students achieving a full score. This result is similar to the findings of a study from Murry and colleagues [17]. In this mixed-methods study, a majority of students received mixed-empathy comments from SPs related to their performance in a medication adherence SP encounter.

It was noteworthy that student recognition of the importance of communication skills positively correlated with performance on the evaluation and the final grade in the communications course. This could be due to additional study and practice time dedicated to the material when it is viewed as important [22,23]. However, it was surprising that a correlation did not exist between the SP ratings of empathy and recognized importance of empathy as a skill. This further explains that, while empathy is viewed as important, this perspective does not necessarily translate to higher performance. This may be because it is a challenging skill to teach, learn, and evaluate. While the practice of communication skills is important overall, practice opportunities that include demonstrating empathy could be particularly beneficial.

While a majority of the students felt the virtual format of the evaluation hindered their confidence and ability to demonstrate non-verbal communication skills, this perception may not correlate with actual performance. Studies comparing performance on virtual and in-person skills-based assessments in pharmacy education demonstrated similar student performance in both environments [24,25]. This is helpful information to support continued use of virtual communication evaluations.

One of the strengths of this study was the use of SPs that were well-trained on using the designated scripts and in evaluating the students based on the supplied rubrics and knowledge of the required communication skills. SPs have demonstrated a positive impact on the development of communication skills and are perceived as providing a more realistic portrayal of a patient encounter compared to internal SPs such as faculty or staff [26,27]. Another strength of the study design is that the students were familiar with the rating criteria after experiencing it in a prior communication evaluation as well as a practice patient counseling evaluation. The students also received SP feedback from the prior communication evaluation, which may give context for the approach to rating these communication soft skills. Limitations of the study include the small number of survey respondents, which consisted of 49% of a single cohort of students within the course. The authors felt it was important to receive feedback from this particular cohort who experienced the communication course in a fully virtual format during the COVID-19 pandemic. For comparison, other cohorts would have experience with both remote and in-person evaluations, which could influence their perceptions of the virtual format.

In this study, the students recognized the importance of developing soft skills and view this as important to future practice. However, the development of communication skills remains a challenge. In a 2013 survey, nearly 30% of respondents indicated a need for growth within their communications-focused curricula. Subsequently, many schools of pharmacy developed and/or revitalized the communications-focused content [28,29,30]. The focus on soft skills remains an important element in teaching the skill of communication. When using lecture-based teaching methods, students may learn what to say but miss the critical element of how to say it [2]. Practice and graded evaluations of communication skills should include key soft skills, such as non-verbal communication and conveying empathy.

## 5. Conclusions

Overall, the ratings of communication skills were similar between the SPs and students’ self-assessment. Student self-assessment is a useful and cost-effective mode of feedback for practice experiences as an alternative to the use of SPs. Additional studies are needed to evaluate if student self-assessment remains comparable to other raters when used as formative feedback.

## Figures and Tables

**Table 1:** Demographics (*n* = 68).

**Age (mean, SD)**	26 years ± 5.03
** Gender (Female; *n*, %) **	47 (69.12)
** Race **	
Asian	20 (29.41)
Asian and White or Caucasian	2 (2.94)
Black or African American	1 (1.47)
White or Caucasian	40 (58.82)
Other	3 (4.41)
Prefer not to answer	2 (2.94)
** Ethnicity **	
Hispanic/Latino	11 (16.18)
Non-Hispanic/Latino	52 (76.47)
Prefer not to answer	5 (7.35)

SD = standard deviation.

**Table 2:** Comparison of the Students’ Self-Assessment and Simulated Patient (SP) Assessment.

Patient Counseling Component	SP Rating (*n*, %)	Self-Assessment Rating (*n*, %)	SP Versus Self-Assessment (*p*-Value) ^a^
**Completed Chunks and Checks**			1.0
Yes	64 (94.1)	62 (91.2)	
No	9 (5.9)	6 (8.8)	
**Demonstrated Empathy**			0.17
Yes	41 (60.3)	41 (60.3)	
Partially	21 (30.9)	26 (38.2)	
No	6 (8.8)	1 (1.5)	
**Non-Verbal Communication**			0.57
Yes	51 (75)	38 (55.9)	
Partially	17 (25)	30 (44.1)	
No	0 (0)	0 (0)	
**Used Patient-Friendly Language**			0.22
Yes	56 (82.4)	57 (83.8)	
Partially	11 (16.2)	11 (16.2)	
No	1 (1.5)	0 (0)	

^a^*p*-value < 0.05 = statically significant.

**Table 3:** Perceptions of Communication Importance (*n* = 68).

Statement	Rating, *n* (%)				
	Strongly Disagree	Somewhat Disagree	Neither Agree nor Disagree	Somewhat Agree	Strongly Agree
**I believe that the development of soft skills, such as empathy, is an important aspect of being a pharmacist.**	0	1 (1.5)	4 (5.9)	7 (10.3)	56 (82.4)
**I recognize the importance of communication skills to my future practice as a pharmacist.**	0	0	2 (2.9)	4 (5.9)	62 (91.2)
**I feel that communication is important to developing patient rapport and trust as a healthcare professional.**	0	0	3 (4.4)	4 (5.9)	61 (89.7)

**Table 4:** Impact of Virtual Learning on Perceived Communication Evaluation Performance by Students.

Impact of the Virtual Format of the Evaluation on Your Ability to…	Rating, *n* (%)		
	Hindered My Ability	Neutral/No Impact	Enhanced My Ability
**Provide Empathy**	28 (41.2)	34 (50)	6 (8.9)
**Develop a Trusting Relationship with the Patient**	31 (45.6)	34 (50)	3 (4.4)
**Demonstrate Non-Verbal Communication**	41 (60.3)	21 (30.9)	6 (8.9)

## Data Availability

The data presented in this study are available on request from the corresponding author. The data are not publicly available due to Family Educational Rights and Privacy Act (FERPA) student privacy protections.

## Supplementary Materials

The following supporting information can be downloaded at: https://www.mdpi.com/article/10.3390/pharmacy10060177/s1, Student Self-Administered Survey.
