# Supplementary material for: Comparison of Students’ Self-Assessment and Simulated Patient Assessment in a Patient Counseling Evaluation and Perceived Importance of Communication Skills
Source: Pharmacy (Basel). 2022 Dec 19;10(6):177. doi: 10.3390/pharmacy10060177 (PMC9787712; doi:10.3390/pharmacy10060177)
Supplement: Supplementary file 1 [file pharmacy-10-00177-s001.zip › pharmacy-2061708-supplementary.pdf]

Supplementary Materials
Student Self-Administered Survey

Demographics

1. What is your name? ______________

2. What is your age in years? ________

3. What gender do you identify with?
    a. Male
    b. Female
    c. Non-binary/other
    d. Prefer not to answer

4. What race do you identify with?
    a. American Indian or Alaska Native
    b. Asian
    c. Black or African American
    d. Native Hawaiian or Other Pacific Islander
    e. White or Caucasian
    f. Other
    g. Prefer not to answer

5. What ethnicity do you identify with?
    a. Hispanic/Latino
    b. Non-Hispanic/Latino
    c. Prefer not to answer

Self-Assessment of Performance in Communication in the Self-Care Counseling Evaluation

After reviewing your evaluation video and reflection upon your performance, how would you self-assess your performance on the following elements of the Self-Care Counseling Evaluation?

Patient Counseling Components	Achievement of Outcome
**Practices Chunks and Checks For Understanding:** Consistently shares info tailored to the other person's needs and capacities, and checks for understanding (ask questions, express doubts) throughout the interaction.	□ Yes<br>□ No
**Demonstrates Empathy:** Recognizes and acknowledges emotional state of self, others, situation expressing care, understanding of the patient's experience and concern verbally and nonverbally.	□ Yes<br>□ Partial<br>□ No
**Appropriate Non-Verbals:** Consistently demonstrates appropriate non-verbal behavior [incl. eye contact, facial expression, posture, position and movement, vocal rate, volume, tone, use of notes and computers without interference, used correct pronunciation, did not use fillers (uh, um) Conveyed confidence].	□ Yes<br>□ Partial<br>□ No

**Used Patient-Friendly Language**<br>Used plain language a patient would likely understand, avoided medical jargon.	☐ Yes<br>☐ Partial<br>☐ No

Perceptions of Personal Communication

Please circle or highlight one of the options below for each question

1. I strongly believe that this course is helping me gain skills that I will need in order to be successful in my career
   Strongly Disagree Disagree Agree Strongly agree

2. I find it challenging to communicate and connect with people.
   Usually Often Sometimes Rarely Never

3. I am often misunderstood.
   Usually Often Sometimes Rarely Never

4. People understand my questions, instructions, and ideas the first time I offer them.
   Usually Often Sometimes Rarely Never

*On a scale from 1 to 5, **one** meaning "very unsatisfied" and **five** meaning "very satisfied" how would you rate your satisfaction with your performance in the encounter, based on the following elements?*

5. Overall communication with standardized patient.

   1 2 3 4 5
   [Very Unsatisfied] [Very Satisfied]

6. Accuracy of clinical information provided.

   1 2 3 4 5
   [Very Unsatisfied] [Very Satisfied]

7. Ability to display empathy.

   1 2 3 4 5
   [Very Unsatisfied] [Very Satisfied]

8. Level of trust established.

   1 2 3 4 5
   [Very Unsatisfied] [Very Satisfied]

9. Depth of understanding regarding the patient's concerns and/or needs.

   1 2 3 4 5
   [Very Unsatisfied] [Very Satisfied]

Perceptions of Communication Importance Overall and in the Profession of Pharmacy

Please circle or highlight one of the options below for each question

1. I believe that the development of soft skills, such as empathy, as an important aspect of being a pharmacist.
   Strongly Disagree Disagree Agree Strongly Agree

2. I recognize the importance of communication skills to my future practice as a pharmacist
   Strongly Disagree Disagree Agree Strongly Agree

3. I feel that communication is important to developing patient rapport and trust as a healthcare professional.
   Strongly disagree Disagree Agree Strongly Agree

In the questions below, please circle the statement in the comparison that you identify with most.

4. I think feelings are important OR I think facts are important

5. It is important to communicate well OR It is important to be knowledgeable

6. I pride myself on being the best OR I pride myself on working hard

7. It is important to be heard OR It is important to listen
